# Supplementary material for: Identification of brain metastasis genes and therapeutic evaluation of histone deacetylase inhibitors in a clinically relevant model of breast cancer brain metastasis
Source: Dis Model Mech. 2018 Jul 6;11(7):DMM034850. doi: 10.1242/dmm.034850 (PMC6078399; doi:10.1242/dmm.034850)
Supplement: Supplementary information [file dmm-11-034850-s1.pdf]

FIRST PERSON

First person – Soo-Hyun Kim

First Person is a series of interviews with the first authors of a selection of papers published in Disease Models & Mechanisms, helping early-career researchers promote themselves alongside their papers. Soo-Hyun Kim is co-first author on 'Identification of brain metastasis genes and therapeutic evaluation of histone deacetylase inhibitors in a clinically relevant model of breast cancer brain metastasis', published in DMM. Soo-Hyun conducted the research in this article while a PhD student in the lab of Normand Pouliot at the Peter MacCallum Cancer Centre, Melbourne, Australia. She is now a Senior Scientist at the SK Pharmaceuticals Cancer Research Center in South Korea, developing and validating tumor mouse models, screening and testing drugs, and investigating mechanisms of tumor metastasis.

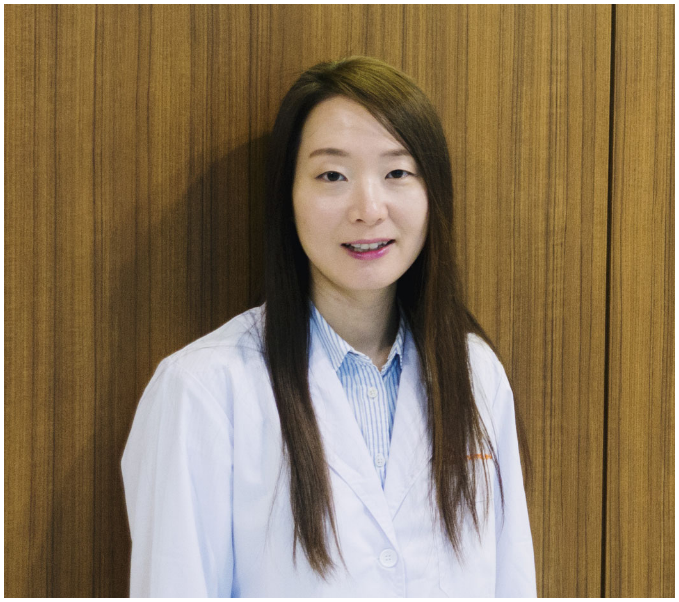

Soo-Hyun Kim

How would you explain the main findings of your paper to non-scientific family and friends?

Breast cancer can travel to other organs through blood vessels in a process called metastasis. While the incidence of brain metastases from breast cancer is increasing, we still know very little about how cancer metastasizes to this organ. Even worse, the presence of a special barrier that safeguards the brain, called the 'blood-brain barrier', blocks most drugs from entering the brain, hence virtually no curative treatments are available to date. This is also is the case for biomarkers used to identify patients likely to develop brain metastases. The mouse model we have developed and presented in this paper allows us to reproduce how breast cancer spreads to the brain under experimental settings that closely resembles progression of the disease in patients. With this model, we tested drugs called histone deacetylase inhibitors, and observed considerable reductions in brain metastases in mice. So we believe that we can use this mouse model to develop treatments for brain metastases and produce useful data for clinical applications.

What are the potential implications of these results for your field of research?

It took a long time to develop this breast cancer brain metastasis mouse model (4T1Br4). Doing so required not only many people's time and efforts but also a significant amount of funding. Against all odds, however, I felt that it was worth investing these resources because this mouse model closely mimics the human disease. In particular, the gene expression profile of 4T1Br4 tumors showed a high level of relevance to that of breast cancer patients with brain metastases. These analyses have taken me towards two potential directions for my future research: first, FCER1G and PECAM1 could be therapeutic targets in breast cancer brain metastasis so investigating these genes is warranted as a potential cure for the disease. Second, the 4T1Br4 model can be used to test various new treatments, including immunotherapy, serving as a useful tool to find the best approach to treat brain metastases.

"I felt that it was worth investing these resources because this mouse model closely mimics the human disease"

Describe what made you transition from academia to industry and what do you do now?

After completion of my PhD, like many other PhD candidates, I was looking for, or dreaming of, a postdoc position in a top-tier lab. But I was told by most labs I contacted that they would only accept self-sufficient postdocs, i.e., having a fellowship, or someone with some years of postdoctoral experience. In the quandary of being an early-career postdoc who had neither self-funding sources nor prior post-doctoral experience, I saw an advertisement posted by a pharmaceutical company who wanted to hire someone able to do molecular biology and mouse work, precisely what I did all the way through my PhD. I realized that I didn't have to stick to academia and I could do what I had experience with, what I was good at and what I loved in industry settings, too. In my current position in industry, I am testing many anti-cancer drugs developed in-house and aiming to generate mouse models for the treatment of brain cancer. If successful, it could be possibly extended to the treatment of breast cancer brain metastasis that was precisely the focus of my PhD research. One of the advantages of being in industry is that I am one step closer to clinical applications, seeing drugs entering clinical trials first-hand.

What changes do you think could improve the professional lives of early-career scientists?

I learned that just having a passion for science would not empower me to keep walking along the academic career path. To survive the early-career crisis, young scientists need funding

Soo-Hyun Kim's contact details: Oncology Research Team, Cancer Research Center, SK Biopharmaceuticals, 221 Pangyoyeok-ro, Seongnam-si, Gyeonggi-do 13494, Republic of Korea.

E-mail: ms.soohyunkim@sk.com

until they have settled in academia. And support for them should be sustained without being threatened by changes in government policies.

What's next for you?

"I dream of my work contributing to saving people's lives and improving the quality of life for patients affected by cancer."

Whether in academia or industry, I will continue working to help patients, those with cancer in particular, by developing and testing new therapies. I am also interested in unravelling the mystery of how cancer metastasizes to other organs, especially to the brain. I dream of my work contributing to saving people's lives and improving the quality of life for patients affected by cancer.

Reference

Kim, S.-H., Redvers, R. P., Chi, L. H., Ling, X., Lucke, A. J., Reid, R. C., Fairlie, D. P., Baptista Moreno Martin, A. C., Anderson, R. L., Denoyer, D. and Pouliot, N. (2018). Identification of brain metastasis genes and therapeutic evaluation of histone deacetylase inhibitors in a clinically relevant model of breast cancer brain metastasis. *Dis. Model. Mech.* **11**: dmm034850.
